# How do *Spondias mombin L* (*Anacardiaceae*) leaves extract increase uterine smooth muscle contractions to facilitate child birth in parturient women?

**DOI:** 10.4314/ahs.v18i2.6

**Published:** 2018-06

**Authors:** Tcha Pakoussi, Aklesso P Mouzou, Kossi Metowogo, Kodjo A Aklikokou, Messanvi Gbeassor

**Affiliations:** 1 Laboratory of Physiology/Pharmacology, Faculty of Sciences, University of Lomé-Togo; 2 Research and Formation Center on Medicinal Plants (CERFOPLAM), University of Lomé-Togo

**Keywords:** *Spondias mombin*, uterus, contractions, calcium

## Abstract

**Background:**

*Spondias mombin L.* (*Anacardiaceae*) leaves were used in Togolese folk to treat dystocia, expel placenta and manage post-partum hemorrhage during child birth.

**Objectives:**

This study aimed to establish how the extract of *S. mombin* leaves increase uterine smooth muscle contractions relevant to its traditional use to facilitate child birth.

**Methods:**

Tests were performed on uterus muscle strips from Sprague-Dawley rats. Central portion of uterine horns were dissected, cleaned of surrounding fat and loose connective tissue, and cut longitudinally into strips which were placed in the organ bath for isometric tension record in presence of different substances.

**Results:**

*S. mombin* leaves extract increased uterine spontaneous contractions. This effect was reduced by indomethacin (2 × 10-6 M), yohimbine (2 × 10-6 M) and 2-aminoethoxydiphenyl borate (2-APB) (5 × 10-5 M), but not by atropine (3.45 × 10-8 M) and cholesterol (2.5 mg/ml).

**Conclusion:**

The pharmacological justification for the traditional use of *S. mombin* leaves to treat dystocia and expel placenta was that its hydro-ethanolic extract induced prostaglandins release, α2-adrenoceptors stimulation, calcium release from internal stores and lifted inhibitory effect of cholesterol on uterine contractions in order to increase uterine smooth muscle contractions.

## Introduction

Uterotonic agents were recognized to increase uterine contractions, not only by intracellular calcium enhancement but also by calcium sensitivity rising at the time of uterine contractile force production during a mechanism involving G protein-coupled receptors^1^. Some plants were reputed to have uterotonic effect and thus allowed calcium mobilization in order to induce uterine smooth muscle contraction. Among them, the fresh leaves of *Spondias mombin* were crushed and used traditionally in Togo by healers to treat dystocia and manage post-partum hemorrhage^2^ and to expel placenta in Nigeria^3^. In our previous study, we have shown that hydro-ethanolic extract of *S. mombin* leaves could contract uterus smooth muscle by cytosolic calcium rising. Calcium mobilization through muscular contraction process is realized by the binding of a ligand to a membrane receptor or to calcium channel or again by membrane depolarization. Membrane receptors implied in the signaling of calcium transduction in the time of smooth muscle contraction were, in many cases, attributed to the stimulation of G protein-coupled receptors (GPCRs)^4^,^5^.

Several membrane receptors of GPCRs group were identified on myometrium membrane and were implied in uterine muscle contraction such as oxytocin receptors^6^, M2 and M3 muscarinic receptors^7^, α1, α2 and β2 adrenoceptors^8^,^9^,^10^, prostaglandin F2α receptors^11^ and estrogenic receptors^12^. Indomethacin is a cyclo-oxygenase inhibitor which blocks prostaglandins synthesis^13^ and uterine contractions^14^. Acetylcholine is an agonist of muscarinic receptors and causes uterine contraction which is blocked by a muscarinic competitive antagonist such as atropine^15^. Yohimbine is a non-selective antagonist of α2 adrenergic receptors^16^. The binding of uterotonic agents to these receptors initiates uterine contraction^4^. Also, the binding of uterotonic agents such as oxytocin, activates phospholipase A2 which induces the production of arachidonic acid. The latter promotes the synthesis of prostaglandins, an important uterotonic agent^17^. 2-aminoethoxydiphenyl borate (2-APB) is an inhibitor of inositol-1,4,5-triphosphate (IP3) receptors and prevents calcium release from sarcoplasmic reticulum^18^,^19^. Cholesterol induced a decrease of uterine smooth muscle contractions and was involved in uterus relative quiescent during pregnancy^20^.

It was shown that methanolic fraction of *S. mombin* leaves extract had uterotonic activity^3^ just as hydro-ethanolic extract^21^. Hydro-ethanolic extract also possessed an estrogenic activity^22^.

The aim of this work was to examine the effects of hydroethanolic extract of *S.mombin* leaves on prostaglandins synthesis or release, calcium release from internal stores and calcium mobilization through adrenergic or muscarinic receptors in order to elucidate its mechanism of action on uterine smooth muscle contractions relevant to its traditional use to facilitate child birth. For that, in vitro tests were performed on rat uterine strips muscle to search the extract effects on this muscle in the presence of indomethacin, yohimbine, 2-APB and atropine.

## Materials and methods

### Plant extraction

*Spondias mombin* leaves were collected in July 2015 in Adéticopé village located 15 Km in north of Lomé city (Togo). The leaves were identified to J.F. Brunel collection and deposited under voucher specimens sample number: TOGO 01851 in Biology and Vegetal Ecology Laboratory herbarium, University of Lomé, Togo.

Leaves (180 g) were washed, dried under air conditioning and were ground. The powder obtained was macerated in water/ethanol 95° mixture in the ratio of 1:1 for 72 hours under intermittent agitation. The mixture was filtered before on cotton and after on filter paper. After filtration, solvent was evaporated by using a rotary vacuum evaporator (R-210 BUCHI) and the extract was obtained as powder. This hydro-ethanolic dry extract yields approximately 21.7% from original dry leaves.

### Animals care and management

Experiments were carried on adult Sprague-Dawley rats weighing 110–180 g. They were bred in Department of Physiology Animal (University of Lome-Togo) where they were housed under standard conditions of light (12 hours' cycles), temperature and tap water ad libitum. This study was conducted in accordance with institutional guidelines and ethics of Laboratory of Physiology/Pharmacology of University of Lome-Togo (ref: 001/2012/CB-FDS-UL). The animals were humanely killed before by anesthetic ether and after by cervical dislocation.

### Drugs and chemicals

#### All chemicals used were reagents obtained from Sigma Chemical Company, St Louis, USA

Soluble cholesterol, Atropine sulfate, Yohimbine hydrochloride and 2-aminoethoxydiphenyl borate (2-APB) were also purchased from Sigma Chemical Company, St Louis, USA. Acetylcholine chloride and Indomethacin were obtained from Abbot, France. All physiological solutions were prepared on the day of the experiment.

### Experimental design

Female rats were mated with male rats to trigged oestrus cycle, and 24 hours after the mating, females were sacrificed by cervical dislocation after ether anesthesis. Central portion of uterine horns were dissected, cleaned of surrounding fat and loose connective tissue, and cut longitudinally into strips (5 mm long, 3 mm wide). Muscle strips were maintained in 10 ml organ baths containing Krebs-Henseleit solution, (mM): NaCl 118; KCl 4.7; CaCl2 2.5; KH_2_PO_4_ 2.5; MgSO_4_ 1.2; NaHCO_3_ 25; glucose 11; pH 7.4. The solution was continuously aerated by Carbogen (95 % O_2_, 5 % CO_2_). The isometric contractile force of the uterine preparations was recorded by using a transducer connected to a recorder (BIOPAC System MP100). The organ was before preloaded (0.5 g) and after an equilibration and a stabilization of spontaneous contractions period of 1 h, the basic tension as well as the spontaneous contractions were continuously recorded. Different volumes of extract were added to the organ bath cumulative manner (5 µl; 10 µl; 15 µl; 20 µl and 25 µl). Those volumes give respectively 0.05 mg/ml; 0.15 mg/ml; 0.30 mg/ml; 0.50 mg/ml and 0.75 mg/ml as final concentration of extract. Thus, the effect of the extract on uterine tissue was studied in presence of indomethacin (2 × 10^−6^ M) or yohimbine (2 × 10^−6^ M) or 2-APB (5 × 10^−5^ M) or atropine (3.45 × 10^−8^ M).

### Data analysis

Data were expressed as mean ± S.E.M. This data was processed by using GraphPad Prism 5 software. Statistical significant of means was determined using Analysis of Variance (ANOVA) followed by Dunnett test. p values smaller than 0.05 were considered to be significant.

## Results

### Effect of extract on uterine smooth muscle in presence of indomethacin

**Figure 1:**
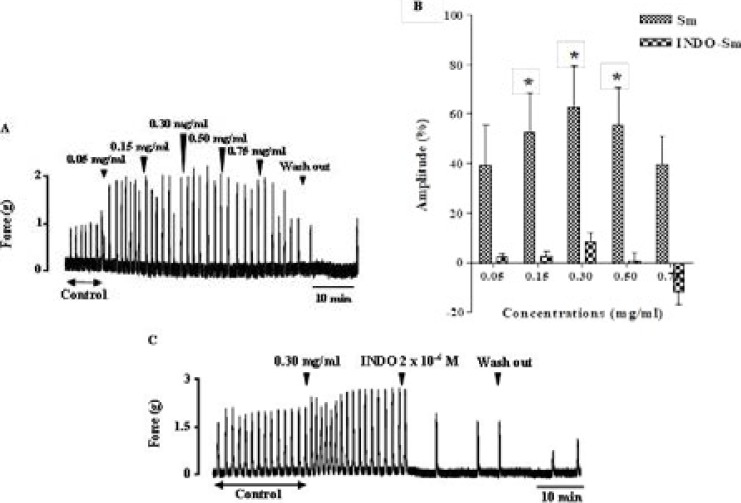
Inhibitory effect of indomethacin on uterus smooth muscle contractions induced by the extract. Values are given as means ± S.E.M. (n = 5) *p < 0.05 (ANOVA followed by Dunnett). Arrow show the time of extract or indomethacin addition to the organ bath followed by the wash out.

The extract induced an increase of uterine spontaneous contraction amplitude (Fig. 1 A). The maximum increase of uterine contractions was observed with 0.30 mg/ml (62.94 ± 16.44 %). The pre-treatment of uterus with 2 × 10-6 M of indomethacin (INDO) was prevented increase in uterine contractions induced by cumulative addition of extract (Sm). Thus, the amplitude of contractions was 2.98 ± 1.62 and 8.31 ± 3.83 % with 0.15 mg/ml and 0.30 mg/ml of extract respectively, compared to the control (Fig. 1 B). Likewise, the increase of uterine spontaneous contractions by 0.30 mg/ml of extract was inhibited by a post-treatment of indomethacin (2 × 10^−6^ M) (Fig. 1 C).

### Effect of extract on uterus pre-treated by yohimbine

**Figure 2:**
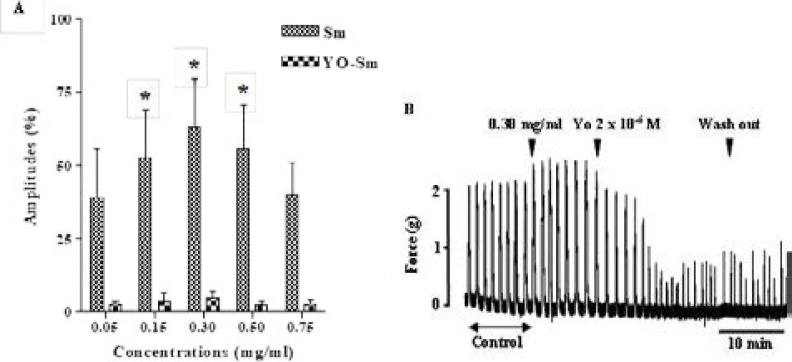
Inhibitory effect of yohimbine on uterus smooth muscle contractions induced by the extract. Values are given as means ± S.E.M. (n = 5) *p < 0.05 (ANOVA followed by Dunnett). Arrow show the time of extract or yohimbine addition to the organ bath followed by the wash out.

The cumulative concentrations of extract didn't augment uterine spontaneous contractions in presence of yohimbine (YO) at 2 × 10^−6^ M. The amplitude of contractions obtained was 3.83 ± 2.76 % and 5.06 ± 2.13 % respectively at 0.15 mg/ml and 0.30 mg/ml of extract in the presence of yohimbine, compared to the positive control (Fig. 2 A). Likewise, the increase of uterine spontaneous contractions by 0.30 mg/ml of extract was reduced by 2 × 10^−6^ M of yohimbine (Fig. 2 B).

### Effect of extract on uterine smooth muscle in presence of 2-APB

**Figure 3:**
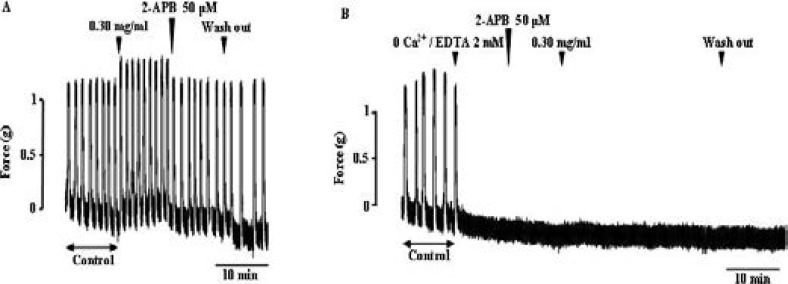
Inhibitory effect of 2-APB on uterus smooth muscle contractions induced by the extract. Arrow show the time of extract or calcium free / EDTA solution or 2-APB addition to the organ bath followed by the

The augmentation of spontaneous contractions of uterine smooth muscle by the extract was reduced by the addition of 50 µM of 2-APB (Fig. 3A). Not only, the amplitude of contractions was reduced but also the basal tone was decreased. When, the uterus strip was mounted in calcium free solution containing 2 mM of EDTA, no contraction of uterine muscle was observed. In these conditions, the extract didn't augment the spontaneous contractions after 2-APB addition (Fig. 3 B).

### Effect of extract on uterine smooth muscle pretreated by atropine

**Figure 4:**
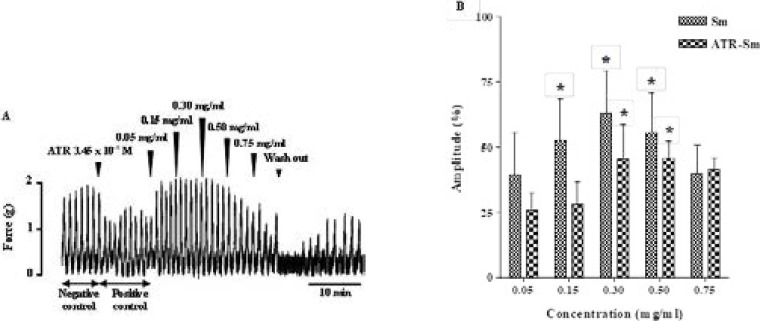
Ineffective effect of atropine on uterus smooth muscle contractions induced by the extract. Values are given as means ± S.E.M. (n = 4) *p < 0.05 (ANOVA followed by Dunnett). Arrow show the time of atropine or extract addition.

Uterine contractions were reduced by 3.45 × 10^−8^ M of atropine (ATR) and increased by cumulative addition of extract (Fig. 4 A). Amplitude of contractions was increased significantly (p < 0.05) with cumulative concentrations of extract in the presence of atropine. We obtained 45.27 ± 13.6 %; 45.67 ± 6.63 and 41.06 ± 4.71 % increase of uterine contractions, respectively, at 0.30 mg/ml; 0.50 mg/ml and 0.75 mg/ml of extract after atropine action, in related to the positive control (Fig. 4 B).

### Effect of extract on uterine smooth muscle in presence of cholesterol

**Figure 5:**
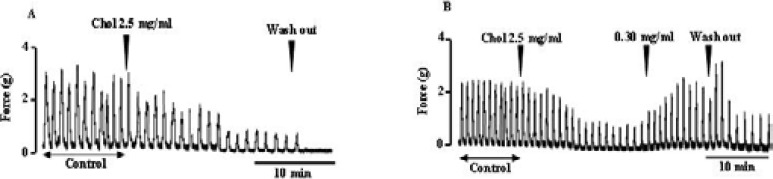
Cholesterol inhibits uterine spontaneous contractions (A) and the extract lifts the inhibitory effect of cholesterol on uterus smooth muscle contractions (B). Arrow show the time of cholesterol or extract addition.

Cholesterol (Chol) at 2.5 mg/ml was significantly reduced (p < 0.01) the amplitude of uterine spontaneous contractions (Fig. 5A). The extract (Sm) at 0.30 mg/ml has lifted this inhibitory effect of cholesterol and induced a significant increase (p < 0.01) of the amplitude of uterine smooth muscle contractions (Fig. 5B). Thus, in the presence of cholesterol, the extract still exerts it effect on uterine smooth muscle.

## Discussion

In the present study, we have shown that hydro-ethanolic extract of *S. mombin* leaves induced a contractile effect on uterus. Thus, the extract possessed uterotonic activity. Some receptors or channels are mediated uterotonic agents action on uterine contractions. Among them, we distinguish prostaglandins, muscarinic, α1and α2-adrenoceptors.

Indomethacin is a cyclo-oxygenase inhibitor which blocks prostaglandins synthesis^13^ and consequently uterine contractions^14^. The reducing of uterine force of contractions in the presence of *S. mombin* leaves extract after a treatment with indomethacin, suggested that the extract contracted uterus by stimulating the synthesis or releasing of prostaglandins. Veale et al.^23^ have observed the same result with *Agapanthus africanus* leaves extract on rat uterus. Indeed, uterine smooth muscle expressed prostaglandins F2α and prostaglandins E2 receptors, which were involved in uterine contractions^24^. The increasing of local production of prostaglandins induces uterine contraction stimulation^5^.

It has been shown that uterine smooth muscle contains two types of adrenergic receptors: α-receptors whose stimulation induces contraction and β2-receptors which, by activation, cause uterine smooth muscle relaxation^9^. Uterus contains particularly α1, α2 and β2 sub-types^9^. Yohimbine is a non-selective antagonist of α2-adrenergic receptors^16^. The addition of yohimbine to the organ bath before or after extract reduced uterine spontaneous contractions. We suggested that the extract goes through α2-adrenoceptors to induce uterine contraction. This result is similar to that of Uchendu and Leek^25^ who have shown that atipamezole, another α2-adrenoceptors antagonist, reduced uterine contractions induced by the root extract of *Dalbergia saxatilis*. This result confirms also our previous study which showed an inhibition of extract effect on uterine smooth muscle by vérapamil. Indeed, Michel et al.^26^ have proved that verapamil was also an antagonist of α-adrenoceptors and, in addition, verapamil is often used as L-type calcium channels blocker^27^.

In our previous study, we have shown that the extract exhibited tonic contraction of uterine muscle in calcium free solution containing EDTA. This suggests that the extract steps in the mobilization of intracellular calcium in the myometrium contraction process^20^. The reduction of the augmentation of spontaneous contractions by the extract in presence of 2-APB and the inability of the extract to augment spontaneous contractions in calcium free solution containing EDTA and 2-APB suggested that the extract acted on IP3 receptors to release intracellular calcium. This result is similar to that of Maruyama et al.^18^ and Morales et al.^19^ which have shown that the addition of 2-APB in calcium free solution containing EDTA abolished spontaneous contractions of the uterus. These authors also explained this effect by the inhibition of IP3 receptors which prevent calcium release from sarcoplasmic reticulum.

Smooth muscle of uterus is rich in cholinergic receptors mainly M3 muscarinic type; acetylcholine is an agonist of this receptor and causes uterine contraction which is blocked by a muscarinic competitive antagonist such as atropine^15^. Abdalla et al.^7^ also proved the expression of M2 and M3 muscarinic receptors in the uterine contraction. The increase of contractile activity of extract was not modified by atropine suggesting that the extract did not bind muscarinic receptors to increase cytosolic calcium. A similar effect was obtained by Eno et al.^28^ who showed that atropine did not block oxytocin effect, an uterotonic agent, on uterine smooth muscle. Acetylcholine enabled to augment cytosolic calcium influx certainly by Voltage Operated Channels (VOCs) and Receptor Operated Channels (ROCs)^29^ and to release calcium from intracellular stock^30^,^31^.

A lipid accumulation in uterine myocytes is responsible of the non expulsion of fetus and of the reducing of uterine sensitivity towards the hormonal stimulation^32^. This study shows that the extract limits the inhibitory effect of cholesterol on uterine contractility. This effect could be explained by the cholesterol depletion on uterine cells membranes by the extract which allows the increase of intracellular calcium in order to augment uterine contractions. Thus, this plant may facilitate the childbirth in obese women.

## Conclusion

Hydro-ethanolic extract of *S. mombin* leaves contracts uterine smooth muscle by its action on force of spontaneous contractions. Prostaglandins synthesis or releasing, α2-adrenergic receptors activation and calcium release from intracellular stores could involve on uterus contraction by the extract in order to increase cytosolic calcium. This study allows us to justify the traditional use of *S. mombin* leaves to facilitate childbirth at the moment of delivery in case of placenta retention.

In further studies, we will try to more understand the inhibitory effect of high concentrations of the extract. By using an oxytocic receptor antagonist such as atosiban, we will show the oxytocic effect of the extract. As yohimbine is also known to act on 5-hydroxytryptamine (5-HT) and dopamine receptors, we will also check out the effect of the extract on these receptors. We will determine the bioactive compound witch is responsible of biological activity of this plant.
